# Emerging Processing Technologies for the Recovery of Valuable Bioactive Compounds from Potato Peels

**DOI:** 10.3390/foods9111598

**Published:** 2020-11-03

**Authors:** Emanuela Calcio Gaudino, Alessandro Colletti, Giorgio Grillo, Silvia Tabasso, Giancarlo Cravotto

**Affiliations:** 1 Department of Drug Science and Technology, University of Turin, Via P. Giuria 9, 10125 Turin, Italy; emanuela.calcio@unito.it (E.C.G.); alessandro.colletti@edu.unito.it (A.C.); giorgio.grillo@unito.it (G.G.); 2 Department of Chemistry, University of Turin, Via P. Giuria 7, 10125 Turin, Italy; silvia.tabasso@unito.it; 3 Centre of Bioanalytical Research and Molecular Design, Sechenov First Moscow State Medical University, 8 Trubetskaya ul, Moscow 119991, Russia

**Keywords:** *Solanum tuberosum*, potato-peel valorisation, phenolic compounds, green extraction, ultrasound-assisted extraction, microwave-assisted extraction

## Abstract

Potato peel (PP) is the major underutilised by-product in the potato-processing industry and a potential source of valuable bioactive molecules. Among them, glycoalkaloids and polyphenols are important precursors for steroid hormones and natural antioxidants, respectively. Moreover, the huge quantities of industrial potato-peel waste that are produced are a rich source of primary metabolites, which principally include starch as well as non-starch polysaccharides, proteins, lipids, lignin and cellulose. All carbohydrates are prone to undergo fermentation to produce ethanol, lactic and acetic acid. Finally, the main portion of PP is made up of alcohol-insoluble matter with a dietary fibre content of approximatively 40%. The present review summarises the recent advances and emerging technologies in potato-peel extraction and further valorisation processing in the food industry.

## 1. Introduction

The potato (*Solanum tuberosum*) is the fourth largest food crop in the world after rice, wheat and maize, and is a very important part of human diets. It was estimated that overall world potato production was 388 million tons in 2017, with more than 40% being produced in China and India (FAO, 2019). This staple crop contains a wide range of molecules with relevant functions in human nutrition, such as vitamins, amino acids and minerals. In particular, nutritional intakes of potassium (up to 693.8 mg/100 g), ascorbic acid (up to 42 mg/100 g), and dietary fibre (up to 3.3%) are provided by several typologies of potato, together with smaller amounts of protein (0.85–4.2%) and other bioactive compounds [1].

The worldwide use of potatoes is increasingly shifting away from fresh and towards machined products, and this leads to huge amounts of potato peel (PP) being produced as industrial waste to be managed. Moreover, the recycling and disposal of this waste poses quite the challenge because of legal restrictions to avoid undesirable consequences such as decomposition with bad smell and being a source of late blight inoculum, leaf roll virus, and other diseases that can spread in neighbouring fields in case of winter field spreading or burial [2].

### 1.1. Chemical Composition of Potato Peel

To fully understand the physicochemical properties of PP, it is crucial to focus on its whole composition (both physical and chemical). The knowledge of these features will support the development of an environmentally friendly approach for the utilisation of PP. Table 1 illustrates the main components [3]. In addition, PP contains various polyphenols and phenolic acids, which are responsible for its antioxidant activities, whereas the fatty acids and lipids show antibacterial properties [4]. The lipid fraction includes triglycerides, alcohols, long-chain fatty acids and sterol esters. Moreover, lignin units have been detected in the cell walls of potatoes [5]. Although PP is rich in starch (52% in dry material), the total amount of fermentable reducing sugar is limited (0.6% on dry material) [6].

#### 1.1.1. Phenolic Compounds in Potato Peel

PP is a great source of phenolic compounds as approximately 50% of these molecules are situated in the peel and adjacent sections [7]. The growing demand of natural antioxidants comes from their applicability as functional ingredients in food formulations as they can ensure the protection of cells against oxidative damage and reduce the risk of oxidative-stress-linked degenerative diseases [8]. For these reasons, the use of by-products to produce food ingredients with excellent nutritional features gained much interest and consequently, their recovery acquired economic attractivity [9]. In this respect, several studies highlighted PP as a source of natural antioxidants [10,11]. These bioactive metabolites can be added to functional foods and can be exploited to produce nutraceuticals by virtue of their possible health benefits [12].

As already mentioned, *Solanum tuberosum* shows an interesting concentration of phenolic compounds that may well integrate the diet. Potato germplasms contain an outstanding variety of polyphenols, in terms of both composition and concentration [13], confirming the presence of active metabolites in all the parts of the tuber [14]. In detail, the detected classes count phenolic acids and flavonoids, including flavanols, flavonols and anthocyanins [3]. A list of phenolic compounds that are present in potatoes can be found in Table 2.

The most common method for the recovery of polyphenols from potatoes is solid–liquid extraction with ethanol, methanol and aqueous alcohol mixtures. However, this approach requires long extraction times and led to moderate yields [15]. Hence, new extraction and isolation techniques have been developed to overcome these issues. Ultrasound-assisted extraction (UAE), microwave-assisted extraction (MAE), and pressurised-liquid extraction (PLE) represent only a limited example of these intensification techniques [14]. PPs contain larger amounts of several nutrients than the pulp; almost 50% of phenolic compounds are found in the skin and adjacent tissues, suggesting that this by-product has a wide range of potential uses [10,15]. PPs have traditionally been used to produce high quality and nutritive animal feeds.

The polyphenol-containing matrix is generally subjected to different types of pre-treatment before the actual extraction step [16]. The main pre-treatments listed in the literature include the physical modification of the biomass (grinding in planetary mills, hammer or blender mills, pre-treatment by ultrasonic or hydrodynamic cavitation and the use of homogenisers), which is useful for increasing mass transport (reduction of the average size of the matrix to be extracted) and the permeability of the material to the extraction solvents (creation of porosity, freeze-drying, cell-wall destruction). However, these processes might be overly harsh for certain compounds, such as in the case of polyphenols, which are labile to different stimuli to varying degrees (temperature, light, etc.) [17]. Desaccharification is a further type of pre-treatment and is used to remove from the matrix components, such as salts and sugars (compounds with little or no activity), that are easily soluble in water at low temperatures. The aim is to enrich the final extract by increasing the activity/dry-extract selectivity without depleting the matrix of polyphenols, the latter of which are normally soluble at temperatures above ambient temperature [18].

#### 1.1.2. Glycoalkaloids in Potato Peel

Unfortunately, the recovery of phenolic compounds, in the case of potato extracts, may entail a significant issue as toxic glycoalkaloids might be concentrated throughout the process [9]. During the germination phase, glycoalkaloids are naturally generated in the tuber, and they can potentially exhibit both adverse and positive effects (acetylcholinesterase inhibition and anticarcinogenic action respectively). PP glycoalkaloid (PGA) content varies according to a number of different factors and conditions (e.g., agrotechnical processes, seasonality, maturation state in the harvest and post-harvest manipulation) and more than eighty different alkaloids have been identified including *alpha*-solanine, *alpha*-chaconine, dehydrocommersonine, atomatine, demissine, dihydro-b-chaconine and dihydrosolanine [19]. Over 330 mg/kg sample concentration, these molecules can cause death [20]. For consumer safety, it is therefore crucial to verify the presence of these metabolites in the final product. In particular, it is recommended that potato tubers should not contain more than 100 mg kg^−1^ fresh weight of these compounds (upper limit of safety: 200 mg kg^−1^ fresh weight) [21]. On the other hand, besides their toxicity and harmful effects, several in vitro and pre-clinical studies have investigated the role that glycoalkaloids may play against many diseases, such as inflammation, glycemia, allergies, microbial infections, fever and even specific types of cancers [22].

For example, a study by Ding *et al.* (1993) has demonstrated the antitumor activity of solasonine, b1-solasonine, solamargine and solanigroside P against MGC-803 cells, and highlighted the possible role of steroidal glycoalkaloids in the treatment of gastric cancer, probably via the downregulation of a p53 mutation, an increase in the Bax-to-Bcl-2 ratio and the activation of caspase-3 to induce apoptosis [23]. For this reason, as reported by Benkeblia (2020), the development of efficient extraction and purification techniques for PGAs, and the enhancement of glycoalkaloid content in PPs via breeding or the molecular engineering of new varieties to increase extraction yield, made PP waste (PPW) a more valuable by-product [22].

#### 1.1.3. Starch, Non-Starch Polysaccharide and Other Valuable Compounds in Potato Peel

The most important chemical components of PPW are starch, non-starch polysaccharides (cellulose, hemicelluloses and pectin), lignin, proteins, lipids and ash [24]. Several different sugars and uronic acids have been identified after sequential extraction, and these include mannose, galacturonic acid, xylose, glucose, fucose, glucuronic acid, galactose, rhamnose and arabinose. During storage, these compounds displayed good stability under acidic conditions, facilitating their further purification and eventual commercialisation or conversion into bioproducts [25]. In this regard, and in light of the growing worldwide energy requirement together with environmental sustainability awareness, carbohydrate waste streams, such as PPW [26], could be a promising alternative for the production of biofuels and chemicals via the biochemical conversion of sugars [27].

In addition, a significant portion of polysaccharides have long been exploited to enhance the texture, water retention and stabilisation of emulsions, and are being ever more frequently integrated into health foods owing to their prebiotic effects and the presence of dietary fibres and mimetic fats [28]. In particular, PP fibres are well known as nutraceuticals in cardiovascular prevention. In fact, many studies have underlined the effects of fibre supplementation in lipid-lowering and as hypoglycaemic agents [29,30,31,32].

Water-soluble polysaccharides that are extracted from PPW are also a promising source of natural antioxidants and can be used as additives in food, pharmaceutical and cosmetic preparations, as highlighted in a study by Jeddou and colleagues [4]. In fact, these bioactive molecules show interesting water-holding and fat-binding capacities in addition to exhibiting a variety of biological activities, including immune-system regulation, inflammation reduction as well as antitumour and antioxidative properties [33].

Lactic acid is an organic acid that can be obtained from PPW. It is widely used in food, pharmaceutical, cosmetic, and industrial applications. Its production generally starts with glucose that is obtained from starch or lignocellulosic biomass that has either undergone separate hydrolysis and fermentation or coupled saccharification and fermentation in the simultaneous presence of enzymes and a pure culture [26].

## 2. Current Strategies for Potato-Peel Valorisation

Simple solid/liquid extraction (SLE) is still the widespread method reported for the extraction of bioactive compounds and in particular, polyphenols from PPs (Table 3). In fact, although its longer extraction times and higher solvent consumption are drawbacks, the equipment utilized is simple and does not require high capital investment. Traditionally, polyphenols are extracted from PPs with organic solvents, such as ethyl acetate, acetone, methanol, and ethanol [34]. Even if these solvents have amazing extraction capacity and a low price, their use has some disadvantages, including high flammability and toxicity (solvent-dependent) [7,35,36]. However, of these organic solvents, ethanol (EtOH) is considered a “GRAS” solvent (generally recognised as safe and harmless) and can therefore be used in the food field [35].

In recent years, the development of new techniques, such as UAE, MAE, pressurised-liquid extraction (PLE) and subcritical water extraction (SWE) (Table 4), for the valorisation of by-products has led to significant reductions in the use of organic solvents, which has improved extraction efficiency and reduced potential toxicity [36,37].

UAE is well known as an efficient unconventional technique for the recovery of several compounds, such as pectin, hemicellulose, polysaccharides, proteins, glycoalkaloids, unsaturated fatty acids and phenolic compounds [38,39]. It is considered to be a versatile, flexible and simple technique that requires relatively small capital investment and is scalable for commercial use [40]. It intensifies extraction by quickening diffusion phenomena and enhancing solvent penetration and mass transfer. UAE has been demonstrated to significantly improve the recovery of polyphenol extracts from PPs, compared to conventional extraction methods alone. Kumari et al. have investigated the UAE, at 33 and 42 kHz, of polyphenols from the PPs of the varieties cream-skinned Lady Claire and pink-skinned Lady Rosetta. Compared to SLE processes alone, the UAE-treated extracts had higher total phenolic content, in particular at lower ultrasonic frequency (33 kHz) better than at higher frequency treatment (42 kHz). The study also highlighted the fact that the Lady Rosetta extract had higher phenolic contents (7.67 mg GAE gdb^−1^ for chlorogenic acid (CGA), as the most representative) and higher antioxidant activity (DPPH value 5.86 mg TE gdb^−1^, FRAP 22.21 mg TE gdb^−1^) than the Lady Claire peel (particularly rich in caffeic acid (CA)). Finally, Peleg’s model of diffusion (R^2^ > 0.92) was found to be a valuable tool with which to understand UAE kinetics and to estimate the extract’s phenolic yield at a variety of extraction time ranges [41]. Although 80% aqueous methanol is the most suitable solvent for the extraction of phenolics from PPs, as was underlined in the aforementioned study, other examinations have shown that water/glycerol mixtures can be very efficient for polyphenol extraction. A study by Paleologou et al. assessed the optimisation of potato-peel extraction and evaluated the extraction efficiency using aqueous mixtures of two bio-solvents, ethanol and glycerol [42,43]. The extractions were assisted by ultrasound (US). The study showed that, under improved conditions, the extraction yields in total polyphenols were 9.11 mg and 8.71 CA equivalents per gram of dry weight, for water/ethanol and water/glycerol mixtures, respectively. The kinetic assay showed the water/ethanol system faster than water/glycerol (diffusion coefficients of 0.46 × 10^−11^ and 0.33 × 10^−11^ m^2^ s^−1^ respectively) [44]. Wang et al. also used UAE (US power 400 W for 4 min, solid-to-liquid mass ratio 1:25, ethanol concentration 80%) to extract potato-peel flavonoids with satisfactory results (maximum extraction yield of flavonoids 2.92%) [45]. These results confirmed those obtained by Samarin et al., in which UAE improved the quantity of total phenolic compounds in the PP extract [34].

Moreover, it is interesting to state that the effects of US power density could deeply influence the extraction of the different polyphenols. According to Alves Filho et al. [46] this technique can be exploited to selectively extract specific caffeoylquinic acids (CQAs) and feruloylquinic acids. In particular, it has also been established how US could promote the hydrolysis of triCQA at 20–50 W/L power density meanwhile that of 3,4-CQA at 50 W/L.

Finally, the strong potential of using UAE in combination with SLE has also been tested on other components, such as some steroidal alkaloids, that are present in the potato-peel waste. Several methods for the extraction of alkaloids from potato have been described, and the most commonly used include polar solvents, such as methanol and ethanol, acid solvents, such as acetic acid, trichloroacetic acid and sulphuric acid, or combined alcohol–acidic solutions [22]. Nevertheless, the use of the UAE with SLE has shown the most promising results and technical efficiency. In particular, a study by Hossain and colleagues identified the optimal UAE conditions using response surface methodology (amplitude: 61 µm, extraction time: 17 min), which resulted in a recovery of 1102 µg steroidal alkaloids/g dried PP compared to 710.51 µg with only SLE. In terms of individual glycoalkaloids, the yields were 273, 542.7, 231 and 55.3 µg/g dried PP for alpha-solanine, alpha-chaconine, solanidine and demissidine, respectively, using UAE [47]. In addition, this technique proved the viability of the concomitant extraction and chemical conversion of alpha-solanine and alpha-chaconine into beta-solanine and beta-chaconine using US [48].

In recent years, Dai et al. [49,50] evaluated MAE as an alternative to conventional methods for the extraction of the bioactive compounds present in PPs [2]. MAE is a novel process that utilises microwave (MW) energy to heat solvents and samples to extract target compounds from the sample into the solvent and can reduce extraction times and solvent consumption as well as promoting higher selectivity towards target molecules [3]. When MWs pass across a biological medium, their energy is absorbed and switched into thermal energy. The capability of a medium to absorb and convert MW energy into heat is defined by its dielectric properties. In a study conducted by Singh et al., MAE was demonstrated to be effective in the extraction of ascorbic acid and selected phenolics, as it used less solvent and considerably reduced the extraction time, although the methanol concentration and extraction time played important roles in the extraction of single phenolics. A maximum total phenolics content of 3.94 mg g^−1^ dry weight was obtained with 67.33% methanol and a MW power level of 14.67% for 15 min. However, the highest contents of ascorbic acid, as well as CA and ferulic acid (FA), were obtained with 100% methanol and a MW power level of 10% for 15 min, while the highest antioxidant activity (evaluated by using the DPPH assay) was obtained under the same conditions, but reducing the treatment time to 5 min [51]. The same research group, in another study, concluded that the yield of the total phenolics extracted during the MAE process was drastically influenced by solvent concentration, extraction time and the dissipation factor of the solvent [52].

Sequential hydrothermal extraction (SeqHTE) is another unconventional technology and is a versatile “green alternative” for repurposing PPs as a resource. It enables the stepwise fractionation of the biomass to extract several bioactive molecules according to the different affinities between water and the compounds at different temperatures. This decreases the residual solid content and thus contributes to mitigating environmental and handling problems. In a recent study by Martinez-Fernandez et al., a SeqHTE process was shown to recover 22.48 and 32.87 mg/g dry peel of polyphenols, and 20–450 and 35–610 mg/kg dry peel of alkaloids from Russet Burbank and peel mixture samples, respectively [53].

Pulsed electric field (PEF)-assisted extraction, a well-known cell-disintegration technique, is based on external electric fields that cause the electroporation of cell membranes, boosting the diffusion of solutes. This permeabilization of cell membranes can be carried out at moderate electric fields (<10 kV/cm) and low specific energies (<10 kJ/Kg). Frontuto et al. have conducted a study to assess the effectiveness of the PEF-assisted extraction, in association with SLE, of total phenolic compounds from both pre-treated (with PEF) and non-pre-treated potato-peel extracts. The results showed that the combination of PEF and SLE granted higher total phenolics yields (10%) and antioxidant activity (9%), compared to the control extraction. In addition, the association of PEF with SLE led to reductions in the duration, temperature and solvent consumption (optimised conditions: 52% ethanol, 230 min and 50 °C for PEF; and 54% ethanol, 233 min and 50 °C for SLE). As highlighted in the study, no significant degradation of polyphenols after PEF (such as chlorogenic, syringic, protocatechuic, caffeic, and *p*-coumaric acids) was revealed by the HPLC–DAD analyses [54]. This interesting result confirms the data reported by Puértolas et al., who investigated the effects of PEF-assisted treatment on the anthocyanin extraction yield from purple-fleshed potato (Solanum tuberosum, variety ‘‘Vitelotte’’) at different extraction times (60–480 min) and temperatures (10–40 C°), using water and ethanol (48% and 96%) as the solvents. In particular, after treatment, it was found that PEF can be performed with water without decreasing the anthocyanin extraction yield from purple-fleshed potato, compared to ethanol (untreated sample using 96% ethanol: 63.9 mg/100 g fw; PEF-treated sample using water: 65.8 mg/100 g fw) [55].

A novel approach, called ohmic heating, has recently been proposed by Pereira et al. It allows water to be used as a solvent for the recovery of phenolics from PPs. By contrast to PEF, ohmic heating applies a constant electric field, and is used as a novel technique for heating foods. Its action is based on the electroporation of cells and their simultaneous heating, which facilitate increased mass transfer into the extracting solvent. Nevertheless, ohmic heating is used less frequently than UAE and PEF because it may degrade thermally labile compounds, although most polyphenols present in PP seem to be heat stable [56].

The importance of green solvents, such as water, and the future perspectives of their use have been highlighted by Singh and Saldana, who examined the application of subcritical water, under high pressure and temperature, to the extraction of polyphenols from PPs. In their study, they registered good recovery rates for phenolic compounds (81.83 mg/100 in 30 min at 180 °C) related to 3 h of extraction with an organic solvent (methanol) [18].

Pressurised liquid extraction (PLE) is a further innovative and “green” technique for the valorisation of by-products. PLE is a technique in which pressure is applied during extraction to allow temperatures above the boiling point of solvents to be used. These higher temperatures increase mass transfer and extraction rates, meaning that PLE generally involves shorter extraction times and lower organic solvent consumption than conventional techniques. Although PLE did not enhance extraction compared to SLE, the use of aqueous ethanol as the extraction solvent, in a recent study, led to the recovery of a higher amount of polyphenols compared to the use of 100% methanol [57]. Hossein et al. have shown that a higher yield of glycoalkaloids was recovered from potato-peel PLE (1.92 mg/g dried PPs) than from conventional SLEn (0.981 mg/g dried PPs). In particular, under two optimum PLE conditions (89% methanol and 80 °C), the levels of individual steroidal alkaloids obtained were 873, 597, 374 and 75 µg/g dried PP for α-chaconine, α-solanine, solanidine and demissidine, respectively. Related values for SLE were 46%, 59%, 40% and 52% lower for α-chaconine, α-solanine, solanidine and demissidine, respectively [58].

## 3. Recent Advances in Potato-Peel Valorisation

Potato is one of the most abundantly produced vegetables in the world, and large quantities of waste are created because of its extensive use in various industries. The peeling process alone can produce 6–10% of the total potato-peel waste, with 0.16 tons of waste produced per ton of processed potato [93]. In the age of the circular economy this waste could represent a real feedstock.

Several studies have demonstrated that it is possible to successfully replace (at least in part) the concentrated feed mixture in sheep and fish rations with potato-peel-based products giving improvements in nutritional parameters, including protein and fat in muscles and liver [94,95]. Potato-peel waste can also be used as biofuels, biofertiliser, biogas and biosorbents after procedures such as fermentation, extraction and others [3]. However, one of the most promising applications for PPs is the production of bioactive compounds. In this regard, phenolic acids, of all the phenolic compounds, have raised great interest as both nutraceuticals and drugs [83]. Gallic acid (GA), chlorogenic acid (CGA), FA, vanillic acid, *p*-coumaric acid (*p*-CUA), CA, protocatechulcgentlsic acid, *p*-hydroxybenzoic acid, syringic acid and salicylic acid are the principle phenolic acids that have been identified in PPs using HPLC [11]. Most of these phenolic substances have been found to present preliminary evidence for antioxidant and anti-inflammatory action in the literature and might be subjects for further study. For example, CGA offers several positive properties, such as antioxidant, antitumoral, anti-inflammatory, antimicrobial, analgesic, neuro- and cardio-protective effects, as highlighted in both in vitro and animal studies [15]. Nevertheless, human randomised clinical trials of potato-peel polyphenols have not yet been performed despite phenolic molecules in PPs being well known, and the number of clinical trials (which have tested these compounds from other food sources) that have documented their potential health applications.

The main limitation to the use and commercialisation of phenolic bioactive compounds that are extracted from PP is the fact that most of the proposed conventional extractive methods are expensive and based on laboratory studies. Thus, the concept of green extraction acquired relevance. This sustainable approach indicates the development of extraction procedures able to reduce energy consumption and providing at the same time a high-quality product. Usually, renewable natural products, alternative energy sources and solvents are the milestones of green extractions [96]. Sustainable extraction would also, theoretically, be much more advantageous in economic terms. However, for this to be true, existing processes must be improved and optimised, and new processes that should also consider using alternative solvents, must be tested [97]. In fact, although the unconventional processes for extracting value-added products are well established in the laboratory, the industrial-scale production with specific cost-effective analyses is still a challenge. In this regard, the uninterrupted availability of PPs and the selective separation of desired components are the major barriers to scale-up. The ideal extraction method for potato-peel polyphenols should be based on: little capital investment, low energy consumption, water as a solvent, high yield and easy integration into current processing lines. Unfortunately, none of the methods described in the literature satisfy all these criteria. In particular, even though significant improvements in extraction efficiency have been obtained using unconventional extraction techniques such as UAE, MWAE and PLE, they still involve high costs compared to chemical methods, and new proposals and solutions to reduce these constraints are, at this moment, still lacking. For this reason, potato processors should adapt the method that best suits their production to optimise extraction yields, sustainability, and high through-put.

Another problem is the high moisture content of PPs, which affects the collection, storage, handling, and transportation. The drying of PPs is essential before any use and an effective dryer for this purpose is important. Even the storage conditions of PPs can influence the antioxidant properties of polyphenols. In a study conducted in Ontario (Canada), the levels of polyphenolic compounds and their antioxidant activity in the PPs were influenced by the storage temperature with highest loss observed at 25 °C, compared to −20.4 °C (minimum loss), which highlights the importance of proper storage conditions in maintaining antioxidant properties [8]. Similar conclusions were made in a study by Lachman et al., who underlined that total antioxidant capacity was modified by both the storage conditions and the potato cultivar. For example, it was reported that cold storage (4 °C) differently influenced the total anthocyanins content of Violette and Highland Burgundy Red cultivars compared to Valfi ones: in the formers, the total antioxidant capacity increased by 18.5% and 12.1%, respectively (if stored at 4 °C instead of room temperature), meanwhile, in the latter, it decreased by 33.9%

## 4. Comparative Potato-Peel (PP) Extraction under Non-Conventional Technologies Using Bio-Based Solvents: A Case Study

**Figure 1:**
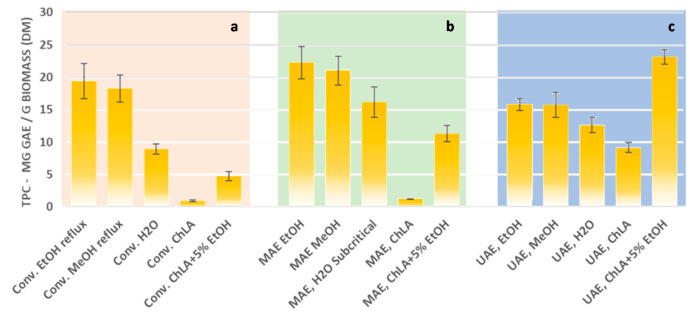
Comparative extractions of Potato-Peel (PP) under conventional (**a**) and non-conventional technologies ((**b**) utilises microwave (MW), (**c**) ultrasound (US)) exploiting sustainable solvents.

The design of sustainable procedures for biomass valorisation (mainly agricultural, industrial, and forest residues) [98,99], using efficient extraction technologies [100], coupled with bio-based solvents [101], is one of the hottest topics in current scientific literature. To partially address this issue, we report herein a comparative, although preliminary, study on potato-peel valorisation under green extraction procedures carried out in our laboratories at the University of Turin. Extractions of *Solanum tuberosum* peels have been carried out in the presence of sustainable solvents (mainly ethanol, water and bio-based solvents) under both conventional and non-conventional technologies (such as US and MW irradiation) in order to identify the best protocol for the recovery of residual bioactive compounds. This preliminary work aimed to demonstrate the synergism that can exist between so-called enabling technologies (MW and US) and bio-based solvents, and that can produce an extract enriched in polyphenols from a food-processing waste benchmark, such as PPs. The general extraction procedures adopted for this comparative study have been described further in Appendix A and draw from previously reported procedures. Three different kinds of solvents have been considered and compared in terms of extraction efficiency (see Figure 1), under both conventional and non-conventional extraction procedures, at a 1:20 S/L ratio: (i) a hydroalcoholic mixture (ethanol or methanol/water 70:30); (ii) distilled water (also applied under subcritical conditions in MAE); and (iii) a choline chloride: lactic acid (1:1) mixture (ChLA).

In the presence of a hydroalcoholic mixture, both the MW and US processes halved the extraction time (from 30 to 15 min), compared to classical extraction conditions (reflux), and almost reached the same value of TPC (total phenolic compounds) extracted. A comparison of the data obtained exclusively using non-conventional processes showed that UAE was found to be much more efficient than MAE. Despite granting a slightly lower quantity of TPC recovery under hydroalcoholic conditions (17 vs. 22 mg GAE/g biomass (dried matrix [DM]), the operating temperatures of UAE were significantly lower than those adopted for MAE (50 vs. 120 °C, respectively, for 15 min extraction time). This confirms the crucial role played by mass transfer, which was significantly enhanced by cavitation during the fast-extractive process under US conditions. Interesting results were only obtained for the application of water as a green extractive solvent under subcritical conditions and MW irradiation (180 °C); good TPC recovery was observed (18 mg GAE/g biomass (DM)).

In addition to conventional hydroalcoholic mixtures and water, a new class of environmentally friendly solvents, namely natural deep eutectic solvents (NaDES), has been explored in potato-peel-extraction experiments. The concept of green solvents is strongly associated with the principles of green chemistry, and NaDESs have recently gained much more consideration than the others available [102], including for use as extraction solvents for phenolic compounds [103]. In brief, a deep eutectic solvent (DES) is a fluid that is usually made of two or three safe and inexpensive components that are capable of self-association, often through hydrogen-bond connections, to create a eutectic mixture with a lower melting point than that of each individual component. Moreover, their production is 100% atom-economic and, unlike ionic liquids (ILs), they are mostly nontoxic and biodegradable. The NaDES ChLA (choline chloride and 1:1 lactic acid) was synthesised and tested in potato-peel extractions under non-conventional conditions and was discussed in this comparative work. Unfortunately, only moderate results were achieved for potato-peel extraction in ChLA under US irradiation (9 mg GAE/g biomass (DM) of TPC). The better results found in UAE, compared to MAE and conventional extractions, can be explained by the boosted mass transfer effect that is induced by cavitation within the viscous extractive mixture due to the presence of NaDES. Better results were achieved by adding a small amount of EtOH (5%) during the ChLA extraction of PP with the best TPC recovery (19 mg GAE/g biomass (DM)) occurring under US in only 15 min of irradiation.

In a typical experiment, 10 g of dry yellow PP (previously milled) was extracted using the proper solvent at a 1:20 S/L ratio: (a) conventional extraction was performed under reflux in a hydroalcoholic mixture at 100 °C using water, and at 120 °C using ChLA; (b) MAE was performed in 15 min at 120 °C (or 180 °C for subcritical water extraction) using a pressurizable MW multimode reactor; (c) UAE was performed in 15 min at 50 °C using an immersion sonotrode working at 21 kHz and 500 W.

Starting from these preliminary results for the US-assisted extraction process, it will be necessary to perform an accurate screening of the most influential extraction parameters in NaDES, such as times, temperatures, matrix/solvent ratios. Moreover, different natural deep eutectic mixtures could be tested for this purpose. In addition, it would also be desirable to conduct a rapid evaluation of synergistic NaDES/extract effects, due to the known stabilizing effects that DES have on extraction products. This comparative study could pave the way for the development of a synergistic process that combines enabling technologies together with green solvents for the recovery of high-added-value products from residual biomass.

## 5. Conclusions

Potato is one of the most abundantly produced vegetables in the world, and large amounts of potato waste are generated because of its widespread use in various industries. The several advantages of potato waste mean that it can serve as the best response for eco-friendly industrial products. One of the most promising applications of PPs concerns its content of polyphenols, which can be extracted using different technologies that are based on the “green chemistry” concept, leading to economic and environmental advantages. However, further investigations are needed to optimise capital investment, energy consumption, the nature of the solvent, yield and integration into current processing lines. To date, none of the unconventional methods described in the literature fulfil all these criteria, and industrial-scale production with specific cost-effective analyses is still a challenge. In addition, the standardisation of cultivation and storage methods is also important to ensure process reproducibility. Finally, there is a strong need for in vitro and in vivo studies to help better understand the pharmacodynamic and pharmacokinetic properties of these bioactive compounds and for the development of new nutraceutical and/or pharmaceutical products.

## Figures and Tables

**Table 1:** Chemical composition of raw potato peel (PP), g per 100 g (Adapted from: Javed et al., 2019 [3]).

Compound	Values Range
Water	83.3–85.1
Protein	1.2–2.3
Total lipids	0.1–0.4
Total carbohydrate	8.7–12.4
Starch	7.8
Total dietary fibre	2.5
Total phenolic content	1.02–2.92
Total flavonoids	0.51–0.96
Ash	0.9–1.6

**Table 2:** Qualitative profile of phenolic compounds in *Solanum tuberosum* (Adapted from: Akyol et al., 2016 [15]).

Hydroxycinnamic acids	- Chlorogenic acid (CGA) Crypto-CGA Neo-CGA - Ferulic acid (FA) - Caffeic acid (CA) - *p*-Coumaric acid (p-CUA)
Hydroxybenzoic acids	- Gallic acid (GA) - Protocatechuic acid (PCA) - Vanillic acid - Salicylic acid
Non-anthocyanin flavonoids	- Catechin (CAT) - EpiCAT - Eriodyctiol - Naringenin - Kaempferol glycosides - Quercetin glycosides
Anthocyanins	- Petunidin glycosides - Malvidin glycosides - Pelargonidin glycosides - Peonidin glycosides
Dihydrocaffeoyl polyamines	- Kukoamine A - *N^1^*,*N^8^*Bis(dihydrocaffeoyl)spermidine - *N^1^*,*N^4^*, *N^12^-*Tris(dihydrocaffeoyl)spermine - *N^1^*,*N^4^*,*N^8^-*Tris(dihydrocaffeoyl)spermidine

**Table 3:** Analytical methods used for phenolic compound extracts from potatoes.

Potato Cultivar	Extract Analysis	Target Class of Compound	Ref.
Cufri chandromukhi	HPLC–DAD	CGA, CA, GA	[59]
9 Italian cultivars	HPLC–UV–Vis	CGA	[60]
Ranger Russet, Norkotah Russet	HPLC–MS	Neo-CGA, CGA, CA, quercetin-3-o-glu-rut, rutin, kaempferol-3-o-rutinoside, cryptoCGA, quinic acid	[61]
23 native Andean cultivars	HPLC–DAD, HPLC–MS, HPLC–FLD	CGA, neo-CGA, crypto-CGA, CA, PCA, vanillic acid, FA, petanin, rutin, kaempferol-3-*O*-rutinoside	[62]
320 specialty potato genotypes	HPLC–DAD	CGA, CA, GA, CAT	[63]
Russet Burbank	Not cited	CGA, FA, vanillic acid, CA, benzoic acid	[64]
Jasim, Atlantic, Jawan, Superior, Jopung	HPLC–MS	CGA, CA, FA, *p*-CUA, *trans*-cinnamic acid	[65]
Nicola, Sieglinde F, Isci 4052, Isci 67	HPLC–DAD	CGA, CA, FA, CAT	[66]
Not cited (Indian cultivar)	HPLC	GA, CA, CGA, PCA	[67]
13 native Andean genotypes	HPLC–DAD	Neo-CGA, crypto-CGA, CGA, kaempferol-3-o-rutinoside, quercetin	[68]
Karlena	HPLC	GA, neo-CGA, PCA, CAT, crypto-CGA, CGA, vanillic acid, CA, FA, *p*-CUA	[69]
Siecle, Purple Majesty, Dakota pearl, FL 1533, Vivaldi, Yukon gold	HPLC–UV–Vis	CGA, CA	[7]
Goldrosh, Nordonna, Dakota pearl, Norkotah, Red Nordland, Sangre, Viking, Dark Red Nordland	HPLC–DAD, HPLC–MS	CGA, CA, GA, FA, CAT, *p*-CUA, o-CUA	[70]
8 cultivars	HPLC–DAD	CGA, CA, epiCAT, p-CUA, vanillic acid, quercetin	[71]
Sava, Bintje	HPLC–DAD	PCA, gentisic acid, GA, CGA, salicylic acid, CA, FA, *p*-CUA	[11]
Bintje, Piccolo, Purple Majesty	HPLC–DAD–MS	CGA, neo-CGA, crypto-CGA, kaempferol rutinose, rutin	[72]
16 cultivars	HPLC–DAD/APCI–MS	CGA, CA, 3-o-CQA, 1-o-CQA	[73]
13 Italian cultivars	HPLC–DAD–MS	5-o-CQA, 4-o-CQA, 3-o-CQA, FA, anthocyanins	[74]
Purple majesty, Yukon gold, Atlantic	UPLC–MS	CGA, CA, FA, sinapic acid	[75]
50 cultivars	HPLC–DAD–MS	CGA, rutin, kaempferol-3-rutinose	[76]
Vitelotte, Luminella, Charlotte, Bintje	UPLC–DAD	CGA, neo-CGA, crypto-CGA, CA, FA, *p*-CUA, syringic acid, vanillic acid, CAT, rutin, kaempferol-3-o-rutinoside	[77]
Sava	HPLC–DAD	GA, PCA, gentisic acid, CGA, vanillic acid, syringic acid, CA, salicylic acid, *p*-CUA, FA	[78]
Not cited	HPLC–DAD	CGA, neo-CGA, crypto-CGA, CUA, genistin, quercetin-3-β-d-galactoside, naringin, naringenin, luteolin, genistein, kaempferol, flavan-3-ol	[79]
Not cited	UPLC–MS	CGA, quinic acid, CA, methyl caffeate	[80]
15 Colombian cultivars	HPLC–DAD–MS	CGA, neo-CGA, crypto-CGA, CA	[81]
Agria	HPLC–UV	CGA, FA, GA	[10]
Valfi, Blaue Elise, Bore Valley, Blue Cango	HPLC–UV	CGA, CA, FA, CUA, crypto-CGA, neo-CGA, *p*-CUA	[82]

HPLC: high performance liquid chromatography; UPLC: ultra performance liquid chromatography; DAD: diode array detector; MS: mass spectrometer.

**Table 4:** Non-conventional extraction methods for phenolic compounds in potatoes.

Potato Cultivar	Extraction System	Experimental Conditions	Target Class of Compound	Ref.
Nicola, Timo, Siikli, Rosamund, Van Gogh	UAE	MeOH and 10% acetic acid (85:15), 30 min	CGA, CA, FA, sinapic acid, vanillic acid, syringic acid	[83]
20 potato cultivars		MeOH (80%), acetic acid (1%), 20 min	CGA, petunidin-3-glucoside chloride, pelargonidin-3-glucopyranoside	[84]
Purple, Innovator, Russet, Yellow		MeOH–acetone–water (7:7:6, *v*/*v*/*v*), 20 min, 30 °C	CGA, CA, p-CUA, FA	[85]
Penta, Marcy		MeOH and 10% acetic acid (85:15), 30 min	CGA, CA, GA, p-CUA, FA	[8]
Diamond		MeOH (70%), ultrasonic water bath with ice, 15 min	CGA, caffeic, 4-hydroxybenzoic, *p*-coumaric, and trans-o-hydroxycinnamic acids	[86]
Russet		Solvents used for extraction: solvent A (25% water, 70% MeOH, 5% acetic acid) solvent B (24% water, 67% EtOH, 9% acetic acid), solvent C (46% water, 51% EtOH, 3% acetic acid), 20min	CGA, CA, neo-CGA	[87]
BP1		MeOH: acetone: ultra-pure water (7:7:1; v:v:v), 5 min	CGA, CA, FA	[88]
Netherlands #7		MeOH (80%) and formic acid (1%), 30 °C, 30 min	GA, PCA, CGA	[89]
Ramus		Continuous air stream ultrasonic bath, 15 min	Total phenolics content	[45]
Calwhite	MAE	EtOH (60%), 80 °C, 2 min, solid-to-solvent ratio 1:40 (g/mL)	CGA, CA, neo-CGA, crypto-CGA, FA, *p*-CUA	[90]
Russt Burbank		MeOH (67.33%), 15 min and a MP of 14.67%	Total phenolics content	[51]
Agria		150–1000 W, 1–7 min	PCA, CGA, neo-CGA, crypto-CGA	[91]
Lady Claire	Pressurized liquid extraction (PLE) + solid–liquid extraction	10.3 MPa, 125 °C, EtOH (70%)	CA	[57]
Red		40 bar, 190 °C, 9 min of static holding time using a flow rate of 3 mL min^−1^	GA, GCA and syringic acid	[92]
Red	Subcritical water extraction	180 °C, 30 min	GA, CGA, CA, PCA, syringic acid, *p*-hydroxyl benzoic acid, FA, CUA	[18]
Vitelotte	PEF aided extraction	3.4 kV/m and 105 µs (35 pulses of 3 µs), water	Anthocyanins	[55]
Vitelotte	Ohmic heating assisted	100 °C for 1 s 200 V/cm, water	Anthocyanins, CGA, FA, ellagic acid, catechin, rutin	[56]
Russet Burbank (dark brown skins)	SeqHTE sequential hydrothermal 3xtraction	Stage 1: 150 or 170 °C; Stage 2: 200 or 220 °C for variable residence times from 10 to 20 min	Total phenolics content (CGA, CA, p-CUA, FA, GA, salicylic acid, catechin, epicatechin, naringenin, syringic acid, and ellagic acid)	[53]

HPLC: high performance liquid chromatography; UPLC: ultra performance liquid chromatography; DAD: diode array detector; UV: ultraviolet detector; MS: mass spectrometer; SeqHTE: sequential hydrothermal extraction, PEF: pulsed electric field; MP: microwave (MW) power (watts).

## Appendix A

### Appendix A.1. Materials and Methods of Comparative Potato-Peel (PP) Extraction under Non-Conventional Technologies Using Bio-Based Solvents

#### Appendix A.1.1. Biomass Material

The *Solanum tuberosum* L. cv. Agria peel used in this work was bought at city market (Turin-Italy). Before use, PP was freeze-dried and milled using a laboratory blender (HGBTWTS360, Waring Blender). Sieving was applied to select <1000 µm granulometry (Giuliani, Italy).

#### Appendix A.1.2. Chemicals

All chemicals were purchased from Sigma-Aldrich and used without further purification. NaDES was obtained via heating: ChLA was prepared with equimolar ratios of choline chloride (ChCl) and lactic acid (LA) [104]. The two components were stirred and heated at 50 °C in a round-bottom flask without adding water until a homogeneous liquid was formed. ChLA was finally collected for biomass extraction without further purification.

### Appendix A.2. General Procedures of Comparative Potato-Peel (PP) Extraction under Non-Conventional Technologies Using Bio-Based Solvents

#### Appendix A.2.1. Conventional Extraction

For the sake of comparison, conventional reflux extraction was performed with a EtOH hydroalcoholic solution (70:30 alcohol/water ratio). The result of this test was used as a benchmark [10]. In a typical extraction, 10 g of dry yellow PP (previously milled) was mixed inside a round-bottom flask with the correct amount of EtOH or MeOH hydroalcoholic solution, at the 1:20 S/L ratio. The mixture was continuously stirred while reflux conditions were reached by means of an oil bath. The extraction was carried out for 35 min. After extraction, the solutions were filtered under vacuum, and fresh extraction solvent was used to thoroughly wash the matrices. The alcoholic fraction was removed using a rotary evaporator, and the crude extracts were then freeze-dried (LyoQuest–85, Telstar, Spain), and the dry material was exploited for total polyphenol content (TPC). For the sake of comparison, the same procedure was applied and the hydroalcoholic solution was replaced with ChLA. All the other parameters were kept unchanged. ChLA solutions were extracted three times with chloroform after the addition of an aliquot of distillate water. The organic fractions were analysed after evaporation. Every test was performed in triplicate and results are reported as an average value ± SD.

#### Appendix A.2.2. Microwave-Assisted Extraction (MAE)

MAE was performed in a SynthWAVE reactor (Milestone Srl, Italy), which is a pressurisable multimode microwave (MW) system that can work under an inert atmosphere (N_2_). Tests were performed by mixing 1 g of dry yellow PP (previously milled) with the desired solvent, at the 1:20 S/L ratio, and then suitable agitation was performed. The protocol was applied to different solvent systems, namely EtOH and MeOH hydroalcoholic solutions (70:30 alcohol/water ratio), deionised water and ChLA NaDES. Before each run, the system was purged with nitrogen three times to reduce oxygen-derived degradations. The reactor was finally pressurised with 20 bar of N_2_ to avoid solvent evaporation at the working temperature. All tests were performed at 1500 W of irradiation with a heating ramp of 5 min. A process temperature of 120 °C was applied for the hydroalcoholic solutions and ChLA, whilst 180 °C was used for subcritical water. The use of a subcritical working temperature has been supported by Singh et al. [18]. The system was stirred at 650 rpm and the temperature was held for 15 min. The MW extraction time was supported by Singh et al. [51]. After extraction, the solutions were filtered under vacuum, and the fresh extraction solvent was used to thoroughly wash the matrices. Where necessary, the alcoholic fraction was removed by a rotary evaporator, and the crude extracts were then freeze-dried (LyoQuest–85, Telstar, Spain) and the dry material was analysed for total polyphenol content (TPC) determination. ChLA solutions were extracted three times with chloroform after the addition of an aliquot of distillate water. The organic fractions were analysed after evaporation. Every test was performed in triplicate and the results were reported as the average value ± SD.

#### Appendix A.2.3. Ultrasound-Assisted Extraction (UAE)

UAE extractions were performed using an immersion sonotrode (HNG-20500-SP, Hainertec Suzhou, China), working at 500 W at a frequency of 21 kHz. Extractions were performed by mixing 5 g of dry yellow PP (previously milled) with the desired solvent at the 1:20 S/L ratio. The mixture was placed in a Pyrex^®^ thimble and cooled by means of an ice bath. The temperature was measured throughout UAE and was maintained under 50 °C to maintain cavitation efficiency [105]. The solution was sonicated for 15 min to avoid overheating. The aforementioned protocol was applied to different solvent systems: hydroalcoholic solutions (70:30 alcohol/water ratio) with EtOH and MeOH, deionised water, and ChLa NaDES. After extraction, the solutions were filtered under vacuum, and fresh extraction solvent was used to thoroughly wash the matrices. Where necessary, the alcoholic fraction was removed using a rotary evaporator, the crude extracts were then freeze-dried (LyoQuest–85, Telstar, Spain) and the dry material was analysed for the total polyphenol content (TPC). ChLA solutions were extracted three times with chloroform after the addition of an aliquot of distillate water. The organic fractions were analysed after evaporation. Every test was performed in triplicate and the results are reported as average value ± SD.

### Appendix A.3. Antioxidant Activity of Comparative Potato-Peel (PP) Extracts Produced under Non-Conventional Technologies Using Bio-Based Solvents

#### Total Phenolic Contents (TPC)

TPC was determined according to previously developed method [106]. The procedure requires a standard curve of GA, used as the reference for phenolic compound quantification. Calibration curve is included between 5 and 250 µg/mL in a H_2_O/DMSO 1:1 mixture. Dried extracts were dissolved in a H_2_O/DMSO 1:1 mixture at a concentration of ~0.8 mg/mL. The GA and analyte (250 µL) were collected into test tubes, together with reactive mixture, as it follows: Folin–Ciocalteu (250 µL, diluted equally with distilled H_2_O), 10% p/v Na_2_CO_3_ solution (500 µL), distilled H_2_O (4 mL). Test tubes were then vigorously shaken and left at room temperature. After 25 min, the absorption of the solutions was measured at 740 nm with a Cary 60 UV–Vis spectrophotometer (1 cm cuvette, Agilent Technologies, Santa Clara, CA, USA). TPC was expressed as GA equivalents (GAE, mg/g) over the dried matrix (DM). All tests were carried out three times and expressed as averages.
